# Resistance to Thyroid Hormone Beta in a Patient Born to a Mother With Undiagnosed Graves’ Disease

**DOI:** 10.1016/j.aace.2023.02.003

**Published:** 2023-02-17

**Authors:** Sujatha Seetharaman, Jose Bernardo Quintos, Federico Salas-Lucia

**Affiliations:** 1 Division of Endocrinology, Department of Pediatrics, The Warren Alpert Medical School of Brown University, Providence, Rhode Island; 2 Section of Adult and Pediatric Endocrinology, Department of Medicine, The University of Chicago, Chicago, Illinois

**Keywords:** pregnancy, thyrotoxicosis, resistance to thyroid hormone, delayed growth, tachycardia

## Abstract

**Background/Objective:**

Graves’ disease is an autoimmune disease associated with high levels of circulating thyroid hormones (THs). Resistance to thyroid hormone beta (RTHβ) caused by mutations in the thyroid hormone receptor beta (*THRB*) gene also can lead to high TH levels. Here, we describe 2 related cases, one of a woman with Graves’ disease, and her newborn with RTHβ.

**Case Report:**

The woman was 27 years of age, with free thyroxine (T4) (FT4) >7.7 ng/dL (0.8-1.8), triiodothyronine of 1350 ng/dL (90-180), and undetectable thyrotropin (TSH), but no symptoms of thyrotoxicosis. She also had thyroglobulin antibodies of 65 (2-38). She was treated with methimazole and atenolol. The newborn neonatal screen showed a TSH of 43 mU/L [upper limit of normal 20 mU/L] and total T4 of 21.8 μg/dL (upper limit of normal 15). At 6 days of age, the newborn had a FT4 of 12.3 ng/dL (0.9-2.3), and unsuppressed TSH. The infant, at 3.5 months of age, was identified to harbor a *THRB* mutation (R438H) inherited from her father, but the brothers and mother had no *THRB* mutation. The newborn had tachycardia and delayed growth and was treated with atenolol and supplemental feeding, resulting in weight gain and reduced heart rate.

**Discussion:**

The perinatal high FT4 and tachycardia could have been influenced by the elevated TH levels of the mother and the fetal RTHβ.

**Conclusion:**

It is difficult to evaluate the etiology of neonatal hyperthyroidism when fetal RTHβ and maternal Graves’ disease are not diagnosed early at birth.

**Highlights**

• This is a rare case of neonatal resistance to thyroid hormone beta with maternal thyrotoxicosis.
• The newborn inherited the mutant thyroid hormone receptor beta allele from the father.
• At birth, the newborn exhibited very high FT4 values, rapidly dropping during the first days of life.
• The infant exhibits symptoms of both thyroid hormone deficiency and excess.
• Treatment with atenolol and supplemental feeding improved her tachycardia and growth delay.

**Clinical Relevance**

In a case of a newborn with resistance to thyroid hormone beta (RTHβ), born to a mother with Graves’ disease, it is difficult to evaluate the differential impact of the fetal RTHβ and maternal Graves’ disease on the neonate’s hyperthyroidism if both conditions are not diagnosed before birth.

## Introduction

Resistance to thyroid hormone is a clinical syndrome of reduced responsiveness of target tissues to thyroid hormone (TH).^1^ The incidence of resistance to thyroid hormone is 1 case per 40 000 live births^2^ and is commonly caused by mutations in the thyroid hormone receptor beta (*THRB*) gene, termed resistance to thyroid hormone beta (RTHβ).^3^ The hallmark of RTHβ is high circulating TH—both thyroxine (T4) and triiodothyronine (T3)^4^—with unsuppressed thyrotropin (TSH).^5^^,^^6^ The clinical presentations are variable, and it is common for RTHβ individuals to have symptoms of TH deficiency and excess (eg, delayed growth and tachycardia).^5^ Herein, we report 2 related cases, 1 of a mother and 1 of her newborn. The mother had undiagnosed Graves’ disease, and had very high levels of circulating TH and antibodies for thyroglobulin but no thyrotoxic symptoms.^7^ The newborn was a female with RTHβ presenting delayed growth, tachycardia, and a mutation in the *THRB* gene inherited from the father. The elevated TH levels of the mother and the fetal RTHβ could have influenced the perinatal high free T4 (FT4) and tachycardia. We conclude that it is difficult to make a differential diagnosis and evaluate the impact of neonatal hyperthyroidism caused by either fetal RTHβ or maternal Graves’ disease if both conditions are not diagnosed before birth.

## Case Presentations

**Fig. 1:**
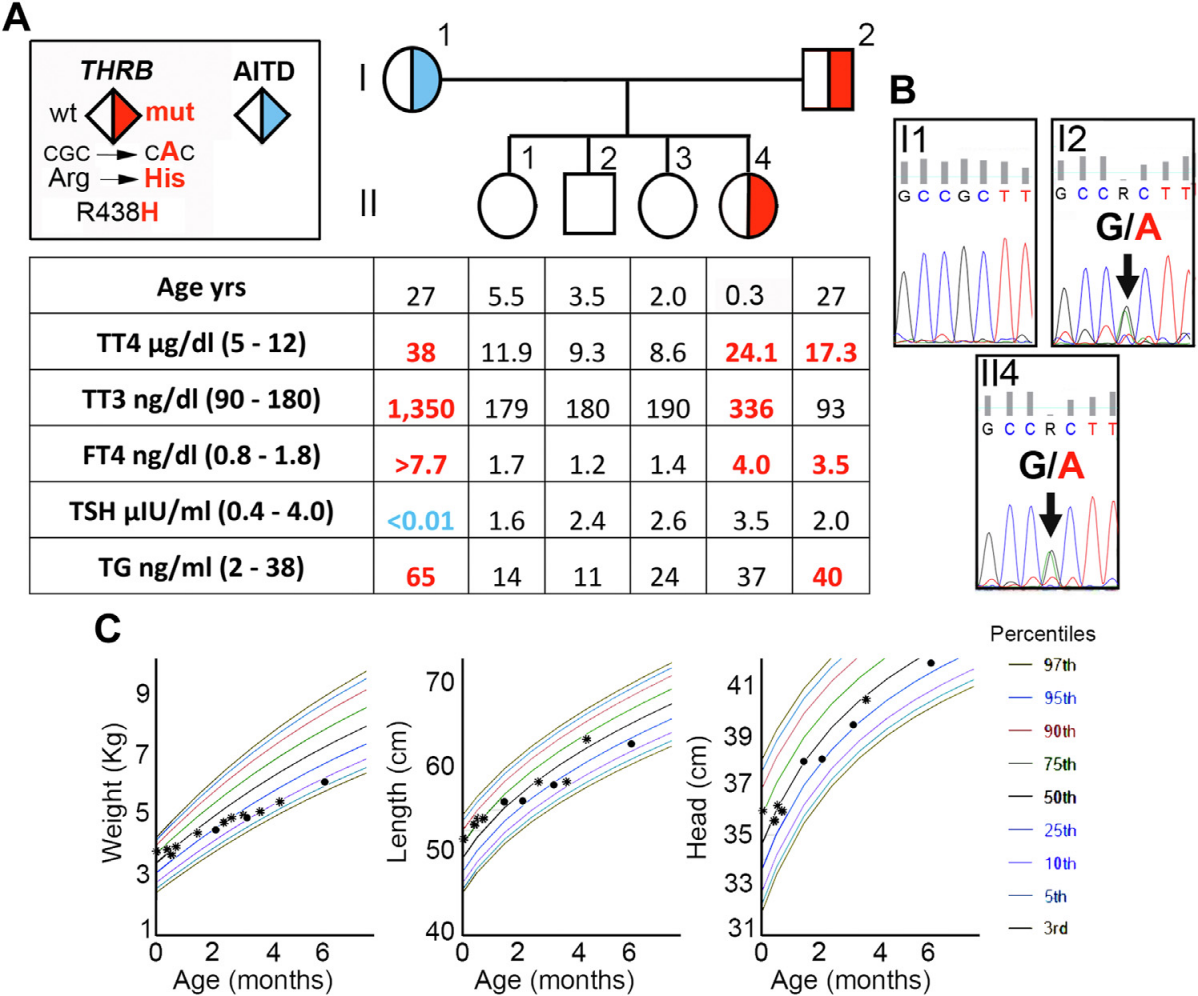
Clinical, biochemical, and genetic data on the infant and her family. *A*, Pedigree of the family and thyroid function test results aligned with each symbol representing a family member. Abnormal values adjusted for age are in bold numbers,^8^ low in blue, and high in red. *B*, Sequencing electropherogram of exon 10 of the thyroid hormone receptor beta gene of representative family members. The father (I-2) and the infant (II-4) harbor a heterozygous guanine-to-adenine transition, replacing the normal arginine 438 with a histidine (R438H). *C*, Growth charts showing the infant’s weight, length, and head circumference during the first 6 months of life. *AITD* = autoimmune thyroid disease; *Arg* = arginine; *CAC* = cytosine adenine cytosine; *CGC* = cytosine guanine cytosine; *FT4* = free T4; *His* = histidine; *Mut* = mutant; *TG* = thyroglobulin; *THRB* = thyroid hormone receptor beta gene; *TSH* = thyrotropin; *TT3* = total triiodothyronine; *TT4* = total tetraiodothyronine; *WT* = wild type.

The first case is a woman of 27 years of age, who exhibited FT4 >7.7 ng/dL (0.8-1.8), total T3 of 1350 ng/dL (90-180), and non-detectable TSH (Fig. 1*A*), but had no symptoms of hyperthyroidism or any manifestation associated with autoimmune diseases, except feeling a little sweaty and having 1 to 3 bowel movements daily with soft stools. She denied jitteriness, racing heartbeats, heat intolerance, weight loss, and feeling short of breath. She denied any history of hypertension and reported unintentional weight fluctuations while in high school, losing 100 lbs. Further evaluation showed positive thyroglobulin antibodies with values of 65 ng/mL (2-38), confirming Graves’ disease, but no additional information about her family history could be obtained. She was treated with methimazole and atenolol but was non-adherent to her medications. She had obesity and 3 previous C-sections. She received routine prenatal care without being checked for thyroid tests. The only thyroid function tests obtained in the woman were after 3.5 months of giving birth to a female newborn (the subject of the second case).

The second case is a female newborn born to unrelated parents at full term via emergency C-section for fetal tachycardia. At birth, the newborn measurements were appropriate for age: weight 3.785 Kg, length 51.5 cm, and head circumference 35 cm (Fig. 1*C*). She had patent ductus arteriosus, meconium aspiration, respiratory distress, and developed pulmonary hypertension, requiring intubation for 12 days. Moreover, she has neonatal thrombocytopenia and received antibiotics for suspected neonatal sepsis.

Her newborn screening at 48 hours showed TSH of 43.2 μIU/mL (upper limit of normal [ULN] 20) and a total T4 of 21.8 μg/dL (ULN 15). A serum sample at 6 days of age showed a dialyzable FT4 of 12.3 ng/dL (2.2-5.3), representing a 232% ULN with an unsuppressed TSH of 15.0 μIU/mL (1-39). Because of the very high FT4 values, her thyroid function tests were monitored weekly (Table). From the second week onward, her FT4 values dropped from 12.3 to an average of 2.7 (0.9-2.3; Table). At 8 weeks, FT4 was 3.7 ng/dL (0.8-1.8), and TSH was 7.7 mU/L (0.35-5.50; high-sensitivity assay). The TSH alpha subunit was 0.3 ng/mL (0.1-0.6), ruling out a TSH-secreting pituitary adenoma.

**Table:** Biochemical Data on the Infant During Her First 10 Months of Life

Age	TSH (μlU/mL)	TT4 (μg/dL)	FT4 (ng/dL)	TT3 (ng/dL)	TSI (IU)	TSH⍺
2 d	43.2 (<20)	21.8 (>5)				
6 d	15.0 (0.5-6.5)		12.3 (0.9-2.3)			
12 d	8.2 (0.5-6.5)		2.8 (0.9-2.3)			
17 d	7.6 (0.5-6.5)		2.6 (0.9-2.3)			
1 mo	6.5 (0.5-6.5)		2.4 (0.9-2.3)			
2 mo	7.7 (0.3-5.5)	23.3 (4.5-10.9)	3.6 (0.8-1.8)	270 (105-245)		0.3 (0.1-0.6)
6 mo	4.8 (0.3-5.0)	19.6 (4.5-12.5)	2.2 (0.7-1.5)	270 (105-245)	<1	
8 mo	4.8 (0.3-5.0)		2.2 (0.7-1.5)	370 (40-1.90)		
10 mo	4.8 (0.3-5.0)		3.9 (0.7-1.5)	327 (40-190)		

FT4 = free T4; TT3 = total triiodothyronine; TT4 = total tetraiodothyronine; TSH = thyroid stimulating hormone; TSH⍺ = thyroid stimulating hormone alpha subunit; TSI = thyroid stimulating immunoglobulin.

During her 3-month visit, while she continued to have a normal thyroid exam with no goiter by palpation (thyroid ultrasound not obtained), no jitteriness, and no proptosis. Her resting heart rate was 160/min (normal range 120-140). Her umbilical cord was still attached that eventually separated at 5 months of age. She also had excessive lanugo hair on her lower back that resolved at 5 months of age. At 3 months of age, her growth was delayed, gaining only 0.4 Kg in 5 weeks (normal weight gain is 2.5 kg during the first 3 months of age), and her weight and linear growth were arrested, going from the 21st and 32nd percentile at 2 months to the 11th and 21st percentile at 3 months, respectively (Fig. 1*C*). Her head circumference decreased from the 51st percentile to the 27th percentile and remained relatively steady afterward (Fig. 1*C*).

We further investigated the infant (at 3.5 months of age) and her family members by obtaining comprehensive thyroid testing and sequencing the *THRB* gene (Fig. 1 *A* and *B*). The infant and her father had an RTHß phenotype (ie, elevated FT4 with unsuppressed TSH) confirmed by identifying a heterozygous missense mutation in exon 10 of the *THRB* gene (R438H; Fig. 1*B*). The father denied any symptoms of hyperthyroidism. Studying the family members of the infant was the reason why we studied the mother (first case), who, while having a marked elevation of circulating serum TH levels, had no *THRB* mutation. The 3 older siblings had serum thyroid tests in the normal range and no *THRB* mutation.

At 6 months of age, the infant had thyroid stimulating immunoglobulin values of <1 IU (normal < 1.3). She had a FT4 of 2.2 ng/dL (0.7-1.5) and total T3 of 270 ng/dL (105-245). After treatment with liquid atenolol (0.5 mg/kg/d) and supplemental feeding between 4 and 6 months of age, the newborn’s weight, body length, and head circumference were in the 21st, 20th, and 33rd percentile (Fig. 1*C*). Her heart rate was 125/min. At 10 months, she had a FT4 of 3.98 ng/dL (0.7-1.5), total T3 of 327 ng/dL (40-190), and a TSH of 4.84 μIU/mL (0.3-5). Her heart rate was 120/min, and she reached age-appropriate developmental milestones.

## Discussion

The woman presented in the first case had very high TH levels but no thyrotoxic symptoms. Thus, it was pertinent to study her *THRB* gene, which showed no mutation, ruling out maternal RTHß.

Individuals with RTHβ due to heterozygous mutations in the *THRB* gene maintain euthyroidism at the expense of high serum TH levels. When pregnant, their high serum TH is congruent with similarly affected fetuses carrying the mutant *THRB* gene. In our second case, the newborn inherited her mutant allele from the father. Under this circumstance, fetal development might be jeopardized because of the exposure to subphysiological TH levels derived from a mother without RTHß.^9^, ^10^ For example, there is a higher prevalence of goiter and short stature in children with RTHß born to normal mothers than those born to affected mothers.^11^ However, the circumstances presented here are atypical. Although the mother had no RTHß, she had very high levels of T4, which could have been congruent with the affected newborn carrying a mutant *THRB* gene.^3^^,^^5^ The normal newborn weight at birth supports this possibility.

Approximately 1% to 5% of neonates born to mothers with Graves’ disease may exhibit transient neonatal hyperthyroidism. If present, the fetus’ clinical manifestations are highly predictive of fetus hyperthyroidism.^12^, ^13^ Tachycardia and goiter are among the main symptoms. Because the newborn had RTHβ, it is unclear whether the high maternal TH levels had an impact during pregnancy or at birth. Indeed, the newborn had a typical case of RTHβ caused by the *THRB* gene mutation R438H. This previously identified mutation decreases the receptor affinity for T3 to 23% of the normal (wild type) receptor (R438H Ka = 0.51 vs wild type Ka = 2.2).^14^ The resulting phenotype is usually hyperthyroid, with unsuppressed TSH. However, the clinical features of both hypothyroidism (growth delay) and hyperthyroidism (tachycardia) are expected.^5^

During her first days of life, the newborn exhibited very high FT4 values that reached ∼534% above the ULN. However, from 12 days of age, the FT4 values were ∼113% above the ULN. A similar drop in FT4 levels was described in a neonate with RTHβ born to a mother with Graves’ disease.^15^ There are 2 possible mechanisms for the increase in TH levels in an infant born to a mother with active Graves’ disease. Passage of TH or thyroid stimulating immunoglobulin across the placenta. However, the rapid drop in the FT4 values suggests that the former was at play, adding to the increased TH levels associated with the RTHB phenotype of the newborn in the first few days of life. Additionally, the newborn at 2 days of life exhibited a TSH of ∼200% ULN, incompatible with the transplacental passage of blocking antibodies, which undetectable TSH usually accompanies. Similarly, the neonatal tachycardia and respiratory distress of the newborn could have been influenced by the transplacental passage of TH and elevated TH levels associated with the RTHβ phenotype of the newborn. Regardless, treatment with atenolol may be prescribed.^16^

This is a rare case of a neonatal RTHß complicated by coincidental maternal uncontrolled Graves’ disease. It can be difficult to evaluate the etiology of neonatal hyperthyroidism when fetal RTHβ and maternal Graves’ disease are not diagnosed early at birth.

## Disclosure

The authors have no multiplicity of interest to disclose.

## References

[1] Refetoff S., DeWind L.T., DeGroot L.J. (1967). Familial syndrome combining deaf-mutism, stuppled epiphyses, goiter and abnormally high PBI: possible target organ refractoriness to thyroid hormone. J Clin Endocrinol Metab.

[2] Lafranchi S.H., Snyder D.B., Sesser D.E. (2003). Follow-up of newborns with elevated screening T4 concentrations. J Pediatr.

[3] Pappa T., Refetoff S. (2021). Resistance to thyroid hormone beta: a focused review. Front Endocrinol.

[4] Salas-Lucia F., Bianco A.C. (2022). T3 levels and thyroid hormone signaling. Front Endocrinol.

[5] Dumitrescu A.M., Refetoff S. (2013). The syndromes of reduced sensitivity to thyroid hormone. Biochim Biophys Acta.

[6] Salas-Lucia F., França M.M., Amrhein J.A. (2022). Severe resistance to thyroid hormone beta in a patient with athyreosis. Thyroid.

[7] Jacobson E.M., Tomer Y. (2007). The genetic basis of thyroid autoimmunity. Thyroid.

[8] Lem A.J., de Rijke Y.B., van Toor H. (2012). Serum thyroid hormone levels in healthy children from birth to adulthood and in short children born small for gestational age. J Clin Endocrinol Metab.

[9] Salas-Lucia F., Pacheco-Torres J., González-Granero S. (2020). Transient hypothyroidism during lactation alters the development of the corpus callosum in rats. An in vivo magnetic resonance image and electron microscopy study. Front Neuroanat.

[10] Anselmo J., Cao D., Karrison T. (2004). Fetal loss associated with excess thyroid hormone exposure. JAMA.

[11] Brucker-Davis F., Skarulis M.C., Grace M.B. (1995). Genetic and clinical features of 42 kindreds with resistance to thyroid hormone. The National Institutes of Health Prospective Study. Ann Intern Med.

[12] Wiersinga W.M., Duntas L., Fadeyev V. (2012). 2012 ETA guidelines: the use of L-T4 + L-T3 in the treatment of hypothyroidism. Eur Thyroid J.

[13] Samuels S.L., Namoc S.M., Bauer A.J. (2018). Neonatal thyrotoxicosis. Clin Perinatol.

[14] Adams M., Matthews C., Collingwood T.N. (1994). Genetic analysis of 29 kindreds with generalized and pituitary resistance to thyroid hormone. Identification of thirteen novel mutations in the thyroid hormone receptor beta gene. J Clin Invest.

[15] Yatsuga S., Hiromatsu Y., Sasaki S. (2012). A two-day-old hyperthyroid neonate with thyroid hormone resistance born to a mother with well-controlled Graves' disease: a case report. J Med Case Rep.

[16] van der Kaay D.C., Wasserman J.D., Palmert M.R. (2016). Management of neonates born to mothers with Graves' disease. Pediatrics.

